# Determination of salivary concentrations of leptin and adiponectin, ability to reduce ferric ions and total antioxidant capacity of saliva in patients with severe early childhood caries

**DOI:** 10.3389/fped.2022.969372

**Published:** 2022-08-31

**Authors:** Bojan Petrović, Nebojsa Stilinović, Ana Tomas, Sanja Kojić, Goran M. Stojanović

**Affiliations:** ^1^Department of Dental Medicine, Faculty of Medicine, University of Novi Sad, Novi Sad, Serbia; ^2^Department of Pharmacology, Toxicology and Clinical Pharmacology, Faculty of Medicine, University of Novi Sad, Novi Sad, Serbia; ^3^Faculty of Technical Sciences, University of Novi Sad, Novi Sad, Serbia

**Keywords:** dental caries, children, biomarkers, saliva, antioxidants, case-control studies

## Abstract

**Introduction:**

One of the most common oral diseases affecting children is early childhood caries (ECC). The link between oxidative stress and ECC has been proven in numerous clinical studies. Technical and biological variability were so high in most of the studies that none of the markers have yet been proven suitable for routine clinical use. This study aimed to evaluate the antioxidant status and the levels of leptin and adiponectin in saliva of children with severe early childhood caries (S-ECC).

**Methods:**

Morning unstimulated saliva samples were collected from children (*n* = 40, 0–6 years old) for the evaluation of oxidative stress which were measured by total antioxidant capacity (TAC), and by the ferric reducing antioxidant power (FRAP) assays, as well as to assess the salivary levels of leptin and adiponectin. FRAP, TAC, leptin and adiponectin concentrations were evaluated in S-ECC group (*n* = 31) and caries free group CF (*n* = 9). All results were analyzed based on age and sex.

**Results:**

Overall median salivary leptin and adiponectin levels were 5.59 pg/mL and 24.86 ng/mL, respectively. Significantly lower leptin levels were observed in saliva of caries free children (4.66 pg/mL) than in the S-ECC group (6.64 pg/mL, *p* < 0.01). No significant difference was observed for adiponectin levels (S-ECC and CF, 25.31 and 23.2 ng/mL, respectively, *p* = 0.961). TAC and FRAP values of saliva had similar values in children with S-ECC and caries free children. TAC and FRAP values also remained stable with the age of the children, without significant differences with respect to sex.

**Conclusion:**

The increased concentrations of leptin in saliva of children with S-ECC suggests that leptin may play a role in inflammatory and immune responses in the development of early childhood caries.

## Introduction

Early childhood caries (ECC), the most common chronic non-communicable disease in preschool children (1, 2), is defined as the presence of one or more carious (uncavitated or cavited) lesions, the absence of one or more teeth (due to caries) or the presence of fillings in any deciduous tooth in a child 72 months of age or younger (2). ECC sometimes has a rapid development and gives early complications in terms of irreversible inflammation of the pulpodentine complex. Severe early childhood caries (S-ECC) is described in the literature using various criteria. For example: “Children who have an “atypical,” “progressive,” “acute,” or “rampant” pattern of dental caries are said to have severe early childhood caries (1, 2),” as well as: “A child with S-ECC may experience severe pain, which could make it difficult for them to eat and speak (3). Also, if the extent of the damage necessitates the extraction of the front teeth by the time the child is 2 or 3 years old, they may experience additional delays in speech articulation and pattern development (4).” S-ECC is also defined as Type III (severe) if ECC was described as “carious lesions affecting almost all teeth including the mandibular incisors (5).” Moreover, S-ECC is described as rampant, Type 3 in children having caries in 14 out of 20 primary teeth, including at least one mandibular incisor (1). Finally, there is a classification of S-ECC based on age (6–13). These proposed terms seem to be gaining international acceptance in the contemporary dental literature (3–7). The number of affected teeth does not always correlate with the intensity of the disease, so indices have been made to objectify the progression of early childhood caries in terms of the development of complications. Monse et al. (14) developed the pufa/PUFA index to assess the pulpo-periapical complications of untreated caries, where visible pulp (P/p), oral mucosal ulceration brought on by root fragments (U/u), a fistula (F/f), or an abscess (A/a) are all scored using this index.

Endogenous antioxidants include compounds that act to reduce oxidative stress and the action of free radicals on the human body (15–17). It is thought that saliva, through salivary antioxidants, might act as the first line of defense against oxidative stress (18, 19). Salivary antioxidants are a group of enzymes including salivary peroxidase, salivary uric acid, and several smaller enzymes. The combined activity of these enzymes in reducing oxidative stress is often referred to as Total Antioxidant Capacity (TAC) of saliva (20, 21).

The connection between ECC, rampant caries and increased TAC of saliva has already been described in the literature. Also, TAC of saliva has been shown to have a linear relationship with age, while no significant dependence has been shown with the respect to sex (21). TAC and Feric Ion Reduction Power (FRAP) have also been studied in patients with periodontitis. Lower TAC and FRAP values in unstimulated and stimulated saliva have been confirmed, however, TAC and FRAP values (7 μmol/mg and 3 μmol/mg, respectively) could not be related to the degree and stage of periodontal disease (22).

Leptin and adiponectin (23) are considered key biomarkers of metabolic dysregulation and comorbidity in both children and adults (24–26). Leptin is a polypeptide hormone of 167 amino acids derived from adipocytes, which has been shown to reduce nutrient intake and increase energy expenditure (27, 28). Serum leptin levels are positively associated with total body fat in adults and children (25, 29). Leptin is a hormone that regulates food intake and energy distribution. It is a protein secreted primarily by fat cells. The salivary glands produce, store and secrete leptin and its level increases with the flow of saliva. A link between leptin levels and tooth loosening during orthodontic treatment has also been found (30). On the other hand, patients with advanced periodontal disease had lower levels of this hormone (31). Its role in other diseases of the oral cavity has not been fully investigated. Adiponectin is an adipocyte-derived hormone that regulates glucose and lipid metabolism, improves fatty acid oxidation and insulin sensitivity, and inhibits glucose production in the liver. In addition, adiponectin has strong anti-inflammatory properties. Other than previously mentioned local effects, adiponectin in breast milk is involved in the regulation of energy balance regulation and may play a role in the regulation of growth and development in the neonatal period and childhood. Adiponectin is also locally produced in salivary glands, with salivary levels significantly correlated with those in plasma. As such, salivary adiponectin sampling has been used as an alternative to blood tests to measure adiponectin levels (31). Having in mind recent increase in connecting ECC with metaboloic disorders, and other diseases in children, we hypothesized that there could be a link between antioxidant status, leptin and adiponectin levels even in young age, since caries is directly related to nutrition. There are reports in literature stating that elevated oxidative stress and inflammation that characterize metabolic syndrome may be caused by dysfunctional adipose tissue, as suggested by a low adiponectin/leptin ratio (32). The rationale was that dental caries could increase the risk of systemic inflammation in a variety of ways (33). For example, among hypertensive pediatric patients with poor oral status, the intensive oxidation of several plasma substrates, increase in reactive metabolites of oxygen, lipid peroxidation, inactivation of prostacyclin and nitric oxide and an imbalance in the total antioxidant capacity were noted (34–36).

The aim of this research was to examine the salivary concentrations of leptin and adiponectin, the ability to reduce ferric ions and the total antioxidant capacity of saliva in patients with early childhood caries, as well as in respondents without tooth decay up to 72 months old.

## Method

### Patients and setting

The prospective clinical case control study included 40 patients under the age of 72 months, at the Clinic for Dentistry of Vojvodina who were referred for examination or therapy with instructions from an ordinance specialist in pediatric and preventive dentistry or an ordinative general dentist in the period from October to December 2021. The study protocol was approved by the Research Ethical Committee of the Dentistry Clinic of Vojvodina in Novi Sad, Serbia and was in accordance with the World Medical Association’s Declaration of Helsinki. In addition, the clinical observation study was approved and registered on the Clin.Trials.gov portal (Determination of Salivary Concentrations of Leptin and Adiponectin, Ability to Reduce Ferric Ions and Total Antioxidant Capacity of Saliva in Patients With Early Childhood Caries LESSEN ClinicalTrials.gov Identifier: NCT05352841). All patients were presented with the opportunity to participate in the clinical trial in writing and asked for written consent. If parents gave written consent and the child oral consent to participate in the study, the usual dental examination (as well as in the cases of non-consent) had been conducted. No additional questionnaire was not used for research purposes, but medical and dental history taking had been taken from patients and parents as a part of standard care. Demographic data, food habits, disease history, fluoride therapy and use of medicines and/or vitamin supplements were detected from general medical documentation. Also, data was taken on whether children have been examined by pediatricians in the last 6 months and whether a normal pattern of growth, nutrition and general health has been established. Growth and nutrition patterns were determined using standard growth charts regularly used during routine clinical assessments. No children were outside the limits of what is considered normal for the respective age based on the perecentiles.

A dental examination was performed during the same visit. Children diagnosed with systemic diseases, who take medication supplements and/or dietary supplements, as well as children with physical or medical disabilities, oral infections, periodontal disease or issues with normal salivary secretion were excluded from the study. A dental examination and a specific dmf index was then performed by a specialist in pediatric and preventive dentistry, and the status of tooth decay was recorded based on the WHO recommendation, 1997 (37). Children were then divided into two groups depending on whether they are diagnosed with severe early childhood caries, S-ECC (at least 6 affected teeth, and PUFA score ≥ 1) (I group) or are without tooth decay lesions CF (II group).

### Sample collection

The prerequisite for taking salivary samples was that the children did not take water and food for 1 h before examination. After examination, unstimulated saliva was collected for analysis (with respect to all conditions in proper sampling and without contamination) in a sterile disposable laboratory container with a wide opening and lid. For saliva collection, depending on age, the child was adequately placed in the dental chair or sit on the parent’s lap. In cooperative, older children, patients were asked to tilt their head slightly and not to swallow or move their tongue or lips during the collection period. Instructions were given to accumulate saliva in the mouth for up to 2 min and he or she were asked to spit accumulated saliva in the admissions cup. About 1 ml of unstimulated saliva was collected and stored at a temperature of 4°C in plastic or glass. In children who were unable to follow instructions due to age and have given oral consent to saliva sampling, unstimulated saliva was collected over a period that also did not last more than 2 min. After coding and anonymisation the samples were immediately relocated to the Department of Pharmacology of the Faculty of Medicine in Novi Sad. Then, the samples were transferred to Eppendorf microepruvets and centrifuged in order to collect supernatant at 3000 RPM for 15 min at 4°C. Each supernatant was then aliquoted into two separate previously coded 0.5 mL tubes. The specimens were kept in the freezer at −20°C until all measurements are carried out.

### Analysis of biomarker levels

The saliva samples were equilibrated to room temperature (18–24°C) prior to ELISA (enzyme-linked immunosorbent assay) analysis. Leptin (ab179884) and adiponectin (ab108786) were measured using commercial human ELISA kits (Abcam, Cambridge, United Kingdom). The assays were carried out according to the manufacturer’s instructions. Each sample was run in duplicate under the same experimental settings. The samples for leptin were analyzed without dilution while they were diluted 2-fold for adiponectin.

The total antioxidant capacity (TAC) of saliva was estimated by the ABTS [2, 2′-Azino-bis(3-ethylbenzothiazoline-6-sulfonic acid)] decolorization assay using microtiter plates. Procedures are based on the method described in Re et al. (38). The assay was standardized with vitamin E hydrosoluble analogue Trolox, thus the total antioxidant capacity was expressed as μg/ml Trolox equivalents. Each sample was analyzed in triplicate.

A ferric reducing antioxidant power (FRAP) assay was performed using a method for microtiter plates described in Choi et al. (39). Briefly, the reaction mixture comprised 152 μL of acetate buffer, 19 μL of TPTZ [2,4,6-Tris(2-pyridil)-S-triazine], 19 μL of FeCl_3_ solution, and 10 μL of the sample. The microplates containing the reaction mixtures were kept in the dark at 37°C for 60 min. Absorbance reading were done at 594 nm using a microplate reader. The assay was standardized with ferrous sulfate heptahydrate, and the results were expressed as Fe^2+^ equivalents (mM). Each sample was analyzed in triplicate.

### Statistical analysis

The number of participants was calculated using G*Power 3.1.9.7 software using *a priori* power analysis for Mann–Whitney test. Input parameters were as follows: effect size *f* value was higher than 0.4; α and β error probabilities were at the same level of 0.10. Allocation ratio was 1:3. The assumptions of central tendency and variation of data were based on the literature survey (26, 40). *Post hoc* analysis showed that based on measured leptin levels as the primary outcome, considering actual sample size, power of 0.799 with the effect size of 0.982 was achieved within alpha level of 0.05.

Data were analyzed using IBM SPSS software, version 23 (IBM Corp., Armonk, NY, United States). Data was assessed for normality using the Kolmogorov–Smirnov test. Descriptive statistics included median, interquartile range, and 25th–75th percentile. The Mann–Whitney *U* non-parametric test was employed to compare the groups. A difference between groups was considered statistically significant for a *p*-value less than 0.05 (*p* < 0.05).

## Results

Of 40 patients that were enrolled in this study, 31 were with severe early childhood caries (S-ECC group) and nine were without caries (CF group). Males were predominant in both groups, 51.61% (16/31) in S-ECC and 55.56% (5/9) in CF group. Median age for S-ECC group was 57.0 [51.0–70.0] while for CF group was 53.0 [46.0–60.5]. No significant differences in age between groups were noticed.

**FIGURE 1:**
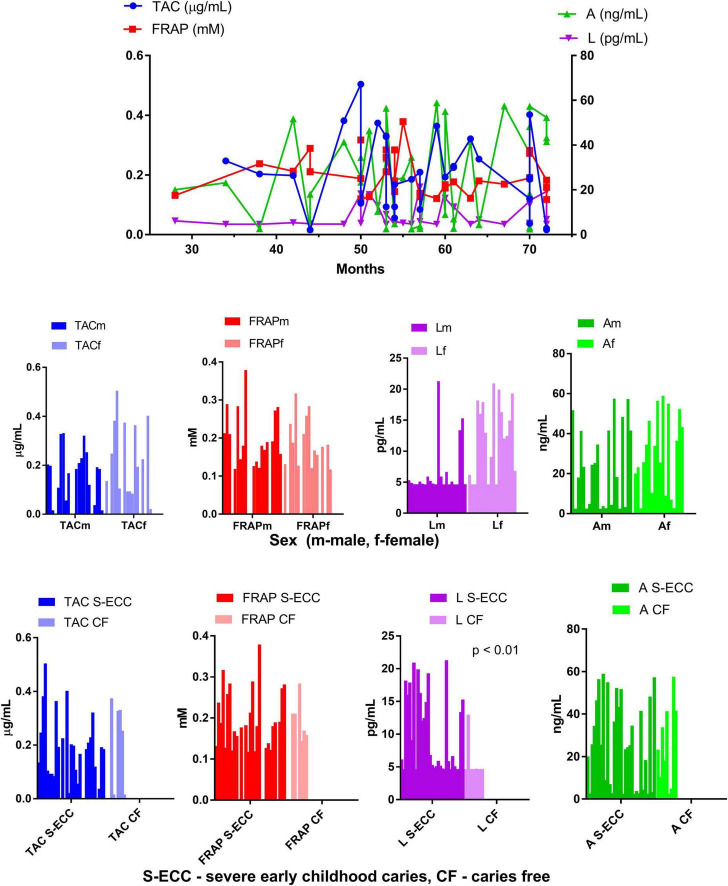
Antioxidant capacity of saliva supernatant measured in patients of different age, sex, and with and without severe early childhood caries.

The results of saliva antioxidant capacity tests (TAC and FRAP) indicated similar values in children with S-ECC and caries free children (Table 1). TAC and FRAP values also remained stable with the age of the children, without significant differences with respect to sex (Figure 1).

**TABLE 1:** Antioxidant capacity of saliva supernatant measured by TAC and FRAP decolorization assays.

Marker	Measurement	Caries (31)	Caries-free (9)	Total	*P*-value*
TAC (μg/ml Trolox equivalents)	Median	0.188	0.290	0.192	0.629
	1st–3rd quartile	0.102–0.234	0.016–0.342	0.096–0.304	
	IQR	0.483	0.358	0.488	
FRAP (mM Fe equivalents)	Median	0.181	0.190	0.181	0.791
	1st–3rd quartile	0.130–0.262	0.155–0.229	0.140–0.253	
	IQR	0.261	0.140	0.261	

*Determined by Mann–Whitney *U* test.

**FIGURE 2:**
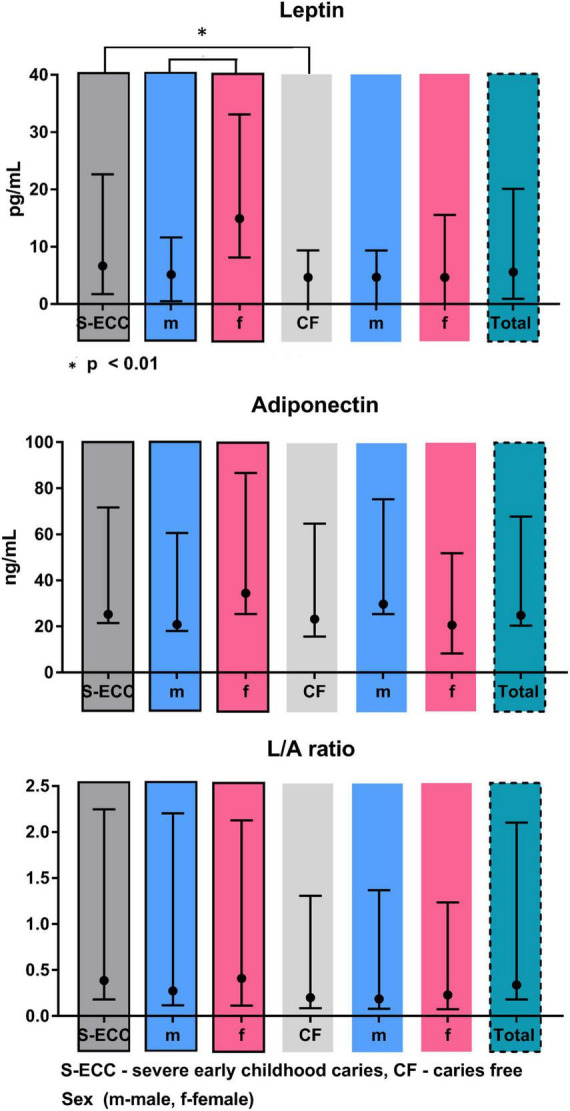
Leptin and adiponectin levels and L/A ration in saliva supernatants of children with or without caries.

Salivary levels of adiponectin and leptin are summarized in Table 2 and Figure 2. Adiponectin level did not differ between groups. On contrary, leptin level was significantly higher in S-ECC group (*U* = 52.0, *p* < 0.01). Moreover, female patients of S-ECC group had significantly higher leptin values than males (*U* = 52.5, *p* < 0.01). A results of Mann–Whitney test for leptin adiponectin ratio (LAR) were close to achieving statistically significant difference (*U* = 70.0, *p* = 0.062).

**TABLE 2:** Concentration of leptin and adiponectin in saliva supernatants of children with or without caries.

Marker	Measurement	Caries				Caries-free				Total	*P*-value*
		Male (16)	Female (15)	Total	*P*-value*	Male (5)	Female (4)	Total	*P*-value*		
Leptin (pg/mL)	Median	5.15	14.92	6.64	<0.01	4.67	4.66	4.66	0.787	5.59	<0.01
	1st–3rd quartile	4.68–6.45	6.82–18.18	4.88–16.01		4.65–4.69	4.65–10.90	4.65–4.71		4.66–14.54	
	IQR	16.63	16.26	16.63		0.09	8.32	8.32		16.63	
Adiponectin (ng/mL)	Median	20.87	34.45	25.31	0.102	29.68	20.61	23.2	0.796	24.86	0.961
	1st–3rd quartile	2.79–39.76	8.99–52.28	3.83–46.37		4.27–45.54	12.28–31.14	7.60–41.46		4.48–42.84	
	IQR	54.81	56.29	56.4		54.87	20.61	54.87		56.4	
L/A ratio	Median	0.273	0.409	0.386	0.752	0.186	0.23	0.20	0.606	0.338	0.326
	1st–3rd quartile	0.155–1.931	0.296–1.716	0.205–1.860		0.105–1.182	0.153–1.004	0.113–1.107		0.158–1.766	
	IQR	5.46	4.52	5.48		1.76	1.11	1.76		5.48	

*Determined by Mann–Whitney *U* test.

## Discussion

Caries is characterized by the same etiological factors that lead to other chronic non-communicable diseases that are associated with increased dietary sugar intake, such as cardiovascular disease, diabetes, and obesity. There is strong evidence that there is a link between oral diseases and chronic systemic disorders (33, 34). Although numerous studies have looked into the links between chronic systemic and dental conditions, it is still unclear how this information can be applied in clinical practice, particularly when it comes to pediatric patients (41–43). More awareness is needed about existing evidence on dental conditions and chronic systemic diseases, potential opportunities for better medical–dental integration in care delivery, and the need for future research on potentially causal links between dental conditions and chronic diseases (44). Early childhood caries is an unacceptable burden for children, families, and society (45). In order to enable the prevention of early childhood caries, it is necessary to actively act on the part of different participants who can influence different aspects of the etiology of caries. Examining the non-specific defense mechanisms of the organism would help shed light on the connection between early childhood caries and other chronic non-communicable diseases, with which they share the same etiological factors. The identification of reliable and specific salivary biomarkers can help with oral disease prevention, diagnosis, and prognosis. Bacteria, DNA, RNA, lipids, metabolites, and proteins are all examples of biomarkers. Rather than focusing on isolated parameter, it is critical to understand the complex interaction between different salivary biomarkers in caries development, progression, and severity (46).

According to a recent meta-analysis, dental caries influences salivary oxidative stress biomarkers, while antioxidant systems are increased in children with dental caries; oxidative damage biomarkers are reduced in saliva of children with dental caries, and salivary parameters are reduced in children with caries lesions (47). Salivary oxidative stress and antioxidant status markers are a promising tool for oral disease research (48). Validation of these biomarkers from the standpoint of confounding and modifying factors is one of the criteria. Two adipokines involved in glucose and lipid metabolism, leptin and adiponectin, have been linked to early childhood growth regulation, energy balance, and metabolic disorders (49).

No significant differences in antioxidant status of saliva in children with and without severe childhood caries (*p* = 0.629 and *p* = 0.791 for TAC and FRAP, respectively) were found in the present study, which is in contrast to other studies who report on a decrease in the total antioxidant potential of saliva in patients with caries (50, 51). However, both of these were conducted on a sample of adolescents (age range 14–18 years) which could explain these observed differences. Also, some studies found a linear relationship between caries severity and TAC (52, 53). This discrepancy could be explained by various response to oxidative stress, where increase in TAC acts as an initial compensatory mechanism, while prolonged oxidative stress may also deplete antioxidant levels, but also due to differences in diet as large share of antioxidants in saliva are food-derived.

It has been reported that dental caries severity increased with higher levels of salivary ghrelin and lower levels of salivary leptin (54). Our results are somewhat different from these findings, as we found higher values of leptin in children with S-ECC (*p* < 0.01). The differences can be attributed in part to the fact that the mentioned study examined older children, 10-year-olds, and also did not take into account the intensity of the disease.

In the study conducted by Martin Gonzales and coworkers it has been shown that the inflamed pulp had significantly higher leptin expression than healthy teeth. The amount of leptin in inflamed pulps was nearly double that of healthy pulps. The presence of leptin in human dental pulp tissues has been demonstrated for the first time in the above mentioned study. The increased expression of leptin in inflamed pulp samples suggests that leptin may play a role in inflammatory and immune responses in the pulp (55). The present study’s findings of a significantly higher relative amount of leptin in inflamed dental pulps support the idea that leptin plays a role in dental pulp inflammatory processes. Our conducted study is in full agreement with the previous statements. It should be borne in mind that the criterion for inclusion in the S-ECC group was the value of the PUFA index was greater than or equal to one, which means that all subjects had at least one tooth with irreversible inflammatory changes in dental pulp.

Silva et al. also investigated the role of TAC and FRAP in S-ECC (56). Their findings point to an S-ECC salivary profile that includes increased salivary proteins, reduced oxidative damage, increased TAC or FRAP and uric acid, and increased superoxide-dismutase activity. Again, the results of our study cannot confirm these findings, because, although not significant, not higher but lower TAC and FRAP values were observed in S-ECC subjects compared to caries free children. Obviously, the reasons for the increased or decreased non-enzymatic and enzymatic antioxidant status in saliva of caries-affected people are unknown. There are suggestions that increased concentrations of uric acid and superoxide-dismutase activity activity may be a compensatory mechanism to reduce oxidative damage, given that their function is to protect oral tissues from the deleterious effects of endogenous or exogenous oxygen or nitrogen reactive species (56).

Salivary carbonyl stress and antioxidant status are influenced by daily variations (57). It has been reported that tooth-brushing and treatment with vitamin C decrease carbonyl stress and increase antioxidant status, and it has been emphasized that these results are important for further research on the role of oxidative and carbonyl stress in the pathogenesis of oral diseases and for the potential use of salivary markers of oxidative and carbonyl stress in the diagnostics of oral diseases, at least on a population level (58). All this should be kept in mind when designing prospective clinical studies, especially when it comes to the youngest participants. In our research, the inclusion criterion was that the child did not eat, drink or brush his or her teeth an hour before the intervention. Also, vitamin therapy was a criterion for exclusion. However, brushing the teeth before visiting the dentist is something that is expected and common. Also, the use of vitamin C is very common in the youngest population. It is certainly necessary to standardize salivary sampling protocols, harmonize procedures and techniques in order to obtain comparable results.

The current study shows that disturbances in salivary leptin levels are linked to early childhood caries, a hormone whose importance has been recognized for several metabolic disorders. This opens up new possibilities for research into leptin as a possible early childhood biomarker. This also adds to mounting evidence that childhood oral infections and inflammation could be linked to the adverse metabolic parameters that manifest later in life.

This study has some limitations. The first is the relatively small number of participants available. Then, it is necessary to have more detailed demographic data on the nutrition and constitution of the respondents, in order to get a more detailed picture of antioxidant status, leptin and adiponectin levels and their possible role in etiopathogenesis, but also the clinical expression of early childhood caries.

## Data availability statement

The raw data supporting the conclusions of this article will be made available by the authors, without undue reservation.

## Ethics statement

The studies involving human participants were reviewed and approved by the Research Ethical Committee of the Dentistry Clinic of Vojvodina in Novi Sad. Written informed consent to participate in this study was provided by the participants’ legal guardian/next of kin.

## Author contributions

BP planned and designed the study. NS and AT carried out the experiments. BP and NS performed the statistical analysis and took the lead in writing the manuscript. BP, NS, AT, and SK contributed to the interpretation of the results. BP and SK designed the figures. GS provided funding and supervision of the project. All authors provided critical feedback, helped shape the research, analysis, and the manuscript, and approved the submitted version.
